# Diet Control and Swimming Exercise Ameliorate HFD-Induced Cognitive Impairment Related to the SIRT1-NF-*κ*B/PGC-1*α* Pathways in ApoE-/- Mice

**DOI:** 10.1155/2023/9206875

**Published:** 2023-03-21

**Authors:** Wei Wei, Zhicheng Lin, PeiTao Xu, Xinru Lv, Libin Lin, Yongxu Li, Yangjie Zhou, Taotao Lu, Xiehua Xue

**Affiliations:** ^1^The Affiliated Rehabilitation Hospital, Fujian University of Traditional Chinese Medicine, Fuzhou, China; ^2^College of Rehabilitation Medicine, Fujian University of Traditional Chinese Medicine, Fuzhou, China; ^3^Fujian Provincial Rehabilitation Industrial Institution, Fujian Provincial Key Laboratory of Rehabilitation Technology, Fujian Key Laboratory of Cognitive Rehabilitation, Fuzhou, China

## Abstract

High-fat diet- (HFD-) induced neuroinflammation may ultimately lead to an increased risk of cognitive impairment. Here, we evaluate the effects of diet control and swimming or both on the prevention of cognitive impairment by enhancing SIRT1 activity. Twenty-week-old ApoE-/- mice were fed a HFD for 8 weeks and then were treated with diet control and/or swimming for 8 weeks. Cognitive function was assessed using the novel object recognition test (NORT) and Y-maze test. The expression of sirtuin-1 (SIRT1), peroxisome proliferator-activated receptor gamma coactivator 1-alpha (PGC-1*α*), brain-derived neurotrophic factor (BDNF), nuclear factor kappa B p65 (NF-*κ*B p65), interleukin-1*β* (IL-1*β*), and tumour necrosis factor-*α* (TNF-*α*) in the hippocampus was measured by western blotting. The levels of fractional anisotropy (FA), *N*-acetylaspartate (NAA)/creatine (Cr) ratio, choline (Cho)/Cr ratio, and myo-inositol (MI)/Cr ratio in the hippocampus were evaluated by diffusion tensor imaging (DTI) and magnetic resonance spectroscopy (MRS) using 7.0-T magnetic resonance imaging (MRI). Our results showed that cognitive dysfunction and hippocampal neuroinflammation appeared to be remarkably observed in apolipoprotein E (ApoE)-/- mice fed with HFD. Diet control plus swimming significantly reversed HFD-induced cognitive decline, reduced the time spent exploring the novel object, and ameliorated spontaneous alternation in the Y-maze test. Compared with the HFD group, ApoE-/- mice fed diet control and/or subjected to swimming had an increase in FA, NAA/Cr, and Cho/Cr; a drop in MI/Cr; elevated expression levels of SIRT1, PGC-1*α*, and BDNF; and inhibited production of proinflammatory cytokines, including NF-*κ*B p65, IL-1*β*, and TNF-*α*. SIRT1, an NAD^+^-dependent class III histone enzyme, deacetylases and regulates the activity of PGC-1*α* and NF-*κ*B. These data indicated that diet control and/or swimming ameliorate cognitive deficits through the inhibitory effect of neuroinflammation via SIRT1-mediated pathways, strongly suggesting that swimming and/or diet control could be potentially effective nonpharmacological treatments for cognitive impairment.

## 1. Introduction

Cognitive impairment has become increasingly commonplace and has been regarded as one of the major health challenges in the past several decades [1], increasing personal, social, and economic burdens. Due to the lack of effective medical therapeutic approaches, patients in advanced stages of cognitive dysfunction are getting worse and not being controlled. Therefore, nonpharmacologic approaches to maintain and improve cognitive decline are drawing increasing attention, with interest in healthy diet and lifestyle behaviours that enhance memory formation [2].

Accumulating evidence has shown that a HFD and less physical activity result directly in cognitive dysfunction [3, 4]. Although cognitive impairment is not the inevitable consequence of an unhealthy lifestyle, early neuroprotection against cognitive dysfunction through therapeutic lifestyle modifications may slow the development of cognitive decline [5]. These findings suggest that a healthy lifestyle, such as physical exercise and diet control, may be the major nonpharmacological means for improving cognitive impairment. The relationship between an unhealthy dietary lifestyle and cognitive impairment is worthy of further discussion.

It has been reported that a HFD harms the ultrastructure and function of the hippocampus and promotes the decline of cognitive function [6, 7], which is closely associated with neuroinflammation [8]. Aberrantly expressed neuroinflammation factors (e.g., TNF-*α* and IL-1*β*) are found in the hippocampus of HFD-fed mice and potentially lead to memory and learning performance loss [9, 10]. The same neuropathological change occurs in individuals with physical inactivity and poor physical performance [11]. Physical activity is an integral part of affecting human growth and overall health [12] and has a positive effect on the regulation of cognitive function [13]. It was demonstrated that aerobic exercise could upregulate the expression of synaptic plasticity-related proteins through inhibitory NF-*κ*B and IL-1*β* release in the hippocampus, suggesting that aerobic exercise might alleviate neuroinflammation to improve cognitive function [14].

SIRT1 is an NAD^+^-dependent class III histone enzyme deacetylase involved in cellular senescence, metabolism homeostasis, neuroinflammation, and ageing by activating PGC-1*α*/BDNF [15] and interrupting the NF-*κ*B pathway [16]. PGC-1*α* is a transcriptional activator and plays a crucial role in mitochondria–related energy metabolism [17]. Activation of PGC-1*α* depends deeply on SIRT1-mediated deacetylation [18]. Deacetylation of PGC-1*α* upregulates the synthesis and secretion of BDNF, a beneficial factor of brain function, to inhibit cognitive decline [19]. Exercise and dietary restriction elevated the expression of PGC-1*α* and BDNF, which contributed to enhancing brain function and improving cognitive decline [20]. In addition, SIRT1 deacetylates histones in the promoter region of NF-*κ*B p65 and inhibits the release of the inflammation-related factors IL-1*β* and TNF-*α*, ultimately reducing inflammation [21, 22]. Our previous study confirmed that swimming exercise and diet control attenuated HFD-induced learning and memory deficits, which was closely associated with high expression of SIRT1 and low expression of NF-*κ*B p65 [23]. Thus, we hypothesized that diet control and/or physical exercise had a positive impact on the improvement in cognitive deficits related to the SIRT1-mediated pathways.

ApoE can effectively regulate the redistribution of lipoprotein and cholesterol. It is the main apolipoprotein of lipid metabolism in the central nervous system and plays an important neuroprotective role. ApoE knockout (ApoE-/-) mice fed a high-fat diet are more prone to metabolic disorders in vivo than wild-type mice. The association of diet control and swimming exercise with high-fat diet-fed ApoE-/- mice and the SIRT1-NF-*κ*B/PGC-1*α* pathway has not been thoroughly investigated. Therefore, this study established the APOE-/- mouse high-fat model to explore the correlation between diet control and swimming exercise and between the high-fat diet ApoE-/- mice and the SIRT1-NF-*κ*B/PGC-1*α* pathway.

## 2. Materials and Methods

### 2.1. Animals

ApoE-/- male mice (aged 20 weeks and weighing 28-34 g) were purchased from Nanjing University-Nanjing Institute of Biomedicine. All mouse experimental protocols were approved by the Animal Management System and Use Committee of Fujian University of Traditional Chinese Medicine (permission number: FJTCMIACUC2020091). The experimental procedures were strictly in accordance with the international animal provisions of the protection and use guidelines. All animals were housed in group cages under a 12 h dark-light cycle at 23 ± 2°C with free access to food and water.

### 2.2. Reagents and Instruments

The instruments and reagents used in these experiments were as follows: a 7.0T animal magnetic resonance instrument (Bruker, Germany); novel object recognition and test system (Boster Bioengineering, China); Y labyrinth video analysis system (Shanghai Xinruan Information Technology, China); high-fat feed (21% fat, 0.15% cholesterol, Jiangsu Medisen Biomedicine, China); isoflurane (Shenzhen Reward, China); immunohistochemistry kit and DAB staining kit (Boster Bioengineering, China); and haematoxylin staining solution and eosin staining solution (Beijing Solebao Technology, China). The primary antibodies in this study were as follows: anti-SIRT1 (Proteintech, USA), anti-TNF-*α* (Proteintech, USA), anti-*β*-actin (Proteintech, USA), anti-IBA1 (Proteintech, USA), anti-GFAP (Proteintech, USA), anti-PGC-1*α* (Abcam, USA), anti-BDNF (Abcam, USA), anti-IL-1*β* (Abcam, USA), NF-*κ*Bp65 antibody (Cell Signaling Technology, USA), goat anti-mouse/anti-rabbit secondary antibody (Proteintech, USA), and antibody diluent (Beijing Biyuntian, China).

### 2.3. Grouping

Experimental grouping was performed as described previously [23]. ApoE-/- mice were randomly divided into 5 groups (5 in each group) as follows: control group (CON), high-fat diet group (HFD), diet control group (DC), swimming exercise group (SE), and diet control + swimming exercise group (DS). After a week of adjustable feeding, the mice in the HFD, DC, SE, and DS groups were fed a high-fat diet (21% fat, 0.15% cholesterol), and the control group was given a normal standard diet for 8 weeks. After that, the HFD group and SE group continued to be fed a high-fat diet, and the remaining groups were switched to a normal standard diet.

### 2.4. Swimming Exercise

The swimming exercise protocol consisted of two phases: adaptation and training [24]. The time-adaptation phase lasted for the first week, and then, all mice were subjected to swimming training for 50 minutes once daily, 6 days per week for a total of 7 weeks. ApoE-/- mice exercised in the round pool (100 × 80 × 60 cm, 35-36°C) for 10 min on the first day of the adaptation phase. The swimming period was extended by 10 min every day until ApoE-/- mice were able to swim for 50 min per day.

### 2.5. NORT

NORT is a simple way to evaluate recognition memory via the difference in the exploration time of novel objects, as described below. The size of the novel object device is an open box with dimensions of 72 × 72 × 25 cm. Three prepared objects, namely, A1, A2, and B, were used, where the size and shape of objects A1 and A2 are the same but different from object B. There are two phases in NORT. In the first phase (acceptance and adaptation phase), each mouse was free to explore for ten minutes in the open box with objects A1 and A2 placed into the symmetric corners on the same side. In the second phase (test phase), A2 was replaced with B, and the mice were allowed to explore freely for 5 min after 1 h and 24 h of habituation. The observation result is expressed as the discrimination index using the following formula: discrimination index = the number of times exploring object B/total number of times exploring objects A1 and B.

### 2.6. Y-Maze Test

Y-maze spontaneous alternations were performed to evaluate short-term memory. The Y-labyrinth apparatus contained three equal-size black arms with a 120° angle between two adjacent arms. The dimensions of each arm were 30 cm in length × 8 cm in width × 15 cm in height. The mice were placed in the intersection of the three arms and allowed to move freely for 8 minutes. The number of times the mouse entered the different arms was continuously recorded. The result was expressed by the spontaneous alternate reaction rate using the following formula: the maximum number of alternations/(total number of arms − 2) × 100%.

### 2.7. MRI

The mice were anaesthetized with 2-3% isoflurane inhalation and placed in a stable and comfortable prone position. The heart rate and body temperature were determined with a detector placed on the abdomen and a thermometer inserted into the rectum. Head coils were applied to fix the mice during the magnetic resonance scan. After adjusting to a suitable position, T2W1 and DTI were performed on the brains of the mice using the RARE sequence. The T2W1 scanning parameters were as follows: TE = 35 ms, TR = 4200 ms, averages = 4, slice thickness = 0.5 mm, and field of view = 20 mm × 20 mm. The EPI program for DTI scanning had the following parameters: TE = 25 ms, TR = 12000 ms, averages = 2, slice thickness = 0.5 mm, and field of view = 20 mm × 20 mm. Finally, MRS scanning was performed. The bilateral hippocampus was selected as the region of interest on the T2-weighted transverse, coronal, and sagittal planes, with a size of 1 mm × 1 mm × 1 mm. After shimming and suppressing water, the FWHM was less than 20 before scanning. The MRS-specific parameters were as follows: TE = 144 ms, TR = 1500 ms, and averages = 256.

### 2.8. Western Blot

Hippocampal tissue was isolated from mouse brains and lysed with RIPA lysis buffer, and the supernatant was obtained and centrifuged at 12,000 × g for 10 min at 4°C. A BCA quantitative kit was used to determine the protein concentration of the obtained supernatant according to the manufacturer's directions. Fifty micrograms of protein from each group was loaded and separated by electrophoresis on 8%, 10%, and 12% SDS–PAGE gels, followed by transfer onto PVDF membranes (0.22 *μ*m). After blocking for 2 h at 25°C with 5% skim milk, the membrane was incubated with antibodies (SIRT1 1 : 1000, PGC-1*α* 1 : 1000, BDNF 1 : 1000, NF-*κ*B 1 : 1000, IL-1*β* 1 : 1000, and TNF-*α* 1 : 1000) overnight at 4°C. On the following day, the membrane was blotted with the corresponding diluent of the secondary antibody for 1 h at 4°C after washing with TBST solution for 3 times. The ultrasensitive ECL luminescent developer was added to the bands, and the data were analysed by Image Lab software.

### 2.9. Statistical Analysis

The data in this study are presented as the mean ± SEM and were analysed using SPSS 22.0 statistical software. One-way ANOVA or *T* test was applied for comparisons among groups. *p* < 0.05 indicated that the differences were statistically significant. All experiments were repeated at least five times.

## 3. Results

### 3.1. Diet Control and/or Swimming Exercise Reduced HFD-Induced Weight Gain

**Figure 1:**
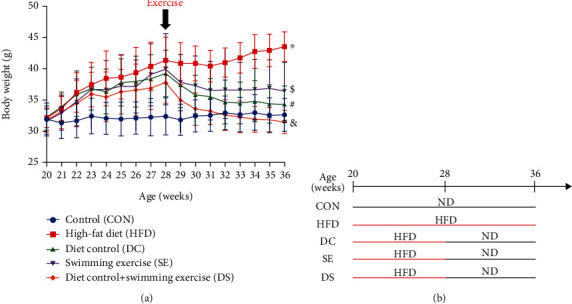
The weight gain trends of ApoE-/- mice in our study. (a) Compared with the control groups, the remaining 4 groups (HFD, DC, SE, and DS groups) significantly increased their body weight after 8 weeks of high-fat diet feeding. Weight gain gradually decreased in the DC, SE, and DS groups from 28 weeks to 36 weeks of age. ^∗^*p* < 0.05 vs. the control group. #*p* < 0.05, $*p* < 0.05, and &*p* < 0.05 vs. the HFD group. (b) Experimental design.

ApoE-/- mice aged 20 weeks were fed a HFD and started diet control and/or received swimming exercise at 28 weeks (Figure 1(b)). The body weight of ApoE-/- mice was measured weekly. Significantly higher body weight gain of mice was found in the HFD group than in the CON group (*p* < 0.05) (Figure 1(a)). The increased body weight gain was gradually reduced at 28 weeks in the DC, SE, and DS groups in contrast to the HFD group (*p* < 0.05) (Figure 1(a)). The lowest body weight gain was found in the DS group (Figure 1(a)).

### 3.2. Diet Control and/or Swimming Exercise Ameliorated HFD-Induced Cognitive Deficits

**Figure 2:**
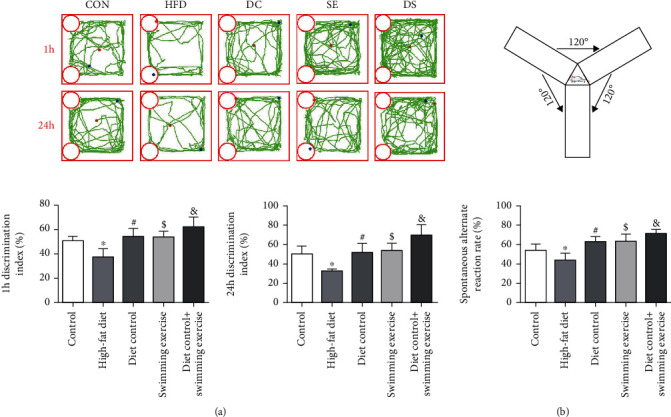
Diet control and/or swimming exercise ameliorated HFD-induced cognitive deficits. Memory performance was assessed by the NORT and the Y-maze test. (a) The 1 h discrimination index (1 h DI) and 24 h discrimination index (24 h DI) were calculated from the NORT data. (b) The spontaneous alternate reaction rate was calculated during the Y-maze test. ^∗^*p* < 0.05 vs. the control group. #*p* < 0.05, $*p* < 0.05, and &*p* < 0.05 vs. the HFD group.

To verify whether diet control and/or swimming exercise contributed to improve HFD-induced cognitive impairment. NORT was used to evaluate recognition memory performance. The HFD group displayed a lower 1 h discrimination index (1 h DI) and 24 h discrimination index (24 h DI) than the CON group (*p* < 0.05) (Figure 2(a)). The 1 h DI and 24 h DI increased gradually in the DC group, SE group, and DS group (*p* < 0.05) (Figure 2(a)). The 1 h DI and 24 h DI of the DS group were higher than those of the SE and DC groups (*p* < 0.05) (Figure 2(a)). The Y-maze test was used to assess working memory (Figure 2(b)). The spontaneous alternate reaction rate of the HFD group was significantly reduced compared with that of the CON group (*p* < 0.05) (Figure 2(b)). The spontaneous alternate reaction rate increased gradually in the DC, SE, and DS groups compared to the HFD group (*p* < 0.05) (Figure 2(b)). The results demonstrated that consumption of a HFD for a long time leads to memory and learning performance loss, and diet control and/or swimming exercise significantly alleviated cognitive decline in ApoE-/- mice.

### 3.3. Diet Control and/or Swimming Exercise Ameliorated the HFD-Induced Decrease in FA in the Hippocampus

**Figure 3:**
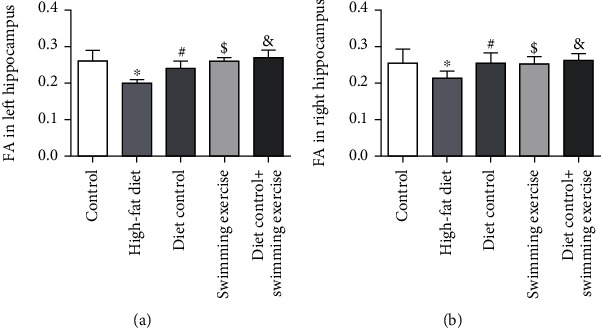
FA levels in the bilateral ApoE-/- mouse hippocampus. (a) FA level in the left ApoE-/- mouse hippocampus. (b) FA level in the right ApoE-/- mouse hippocampus. ^∗^*p* < 0.05 vs. the control group. #*p* < 0.05, $*p* < 0.05, and &*p* < 0.05 vs. the HFD group.

It has been demonstrated that DTI is a novel MRI technique in the hippocampus to detect the impact of damage on white matter structural integrity, which is closely correlated with cognitive decline. High FA indicates that the white matter tracts are intact. In this study, a significant decrease in FA levels was found in the bilateral HFD-fed ApoE-/- mouse hippocampus compared with the CON group (*p* < 0.05) (Figures 3(a) and 3(b)). There was a remarkable upward trend of FA in the DC, SE, and DS groups (*p* < 0.05) (Figures 3(a) and 3(b)). The data suggested that diet control and/or swimming exercise significantly alleviated cognitive decline in ApoE-/- mice by maintaining the structural integrity of white matter in the hippocampus.

### 3.4. Diet Control and/or Swimming Exercise Ameliorated HFD-Induced Neurometabolic Abnormalities in the Hippocampus

**Figure 4:**
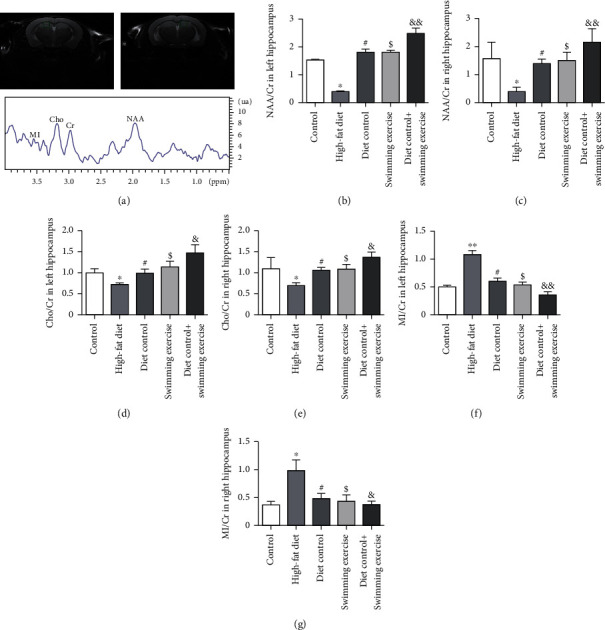
(a) NAA/Cr, Cho/Cr, and MI/Cr ratios in the bilateral ApoE-/- mouse hippocampus. (b, c) NAA/Cr ratio in the bilateral hippocampus of ApoE-/- mice. (d, e) Cho/Cr ratio in the bilateral ApoE-/- mouse hippocampus. (f, g) MI/Cr ratio in the bilateral ApoE-/- mouse hippocampus. ^∗^*p* < 0.05 vs. the control group. #*p* < 0.05, $*p* < 0.05, &*p* < 0.05, and &&*p* < 0.01 vs. the HFD group.

Structural-metabolic variations in the hippocampus are responsible for the initiation of cognitive dysfunction. To clearly explore the alterations in metabolites, magnetic resonance spectroscopy was applied to evaluate NAA/Cr, Cho/Cr, and MI/Cr (Figure 4(a)). There were significant decreases in NAA/Cr and Cho/Cr and an increase in MI/Cr in the bilateral HFD-fed ApoE-/- mouse hippocampus compared with the CON group (*p* < 0.05) (Figures 4(b)–4(g)). NAA/Cr and Cho/Cr were higher in the DC group, SE group, and DS group than in the HFD group (*p* < 0.01, *p* < 0.05) (Figures 4(b)–4(e)). Moreover, MI/Cr decreased gradually in the DC, SE, and DS groups in contrast to that in the HFD group (*p* < 0.01, *p* < 0.05) (Figures 4(f) and 4(g)). These results demonstrated that the neurometabolic abnormalities of the hippocampus in HFD-fed ApoE-/- mice were strongly associated with cognitive impairment, which could be improved by diet control and/or swimming exercise.

### 3.5. Diet Control and/or Swimming Exercise Inhibited HFD-Induced Neuroinflammation in the ApoE-/- Mouse Hippocampus through the SIRT1-Mediated Pathway

**Figure 5:**
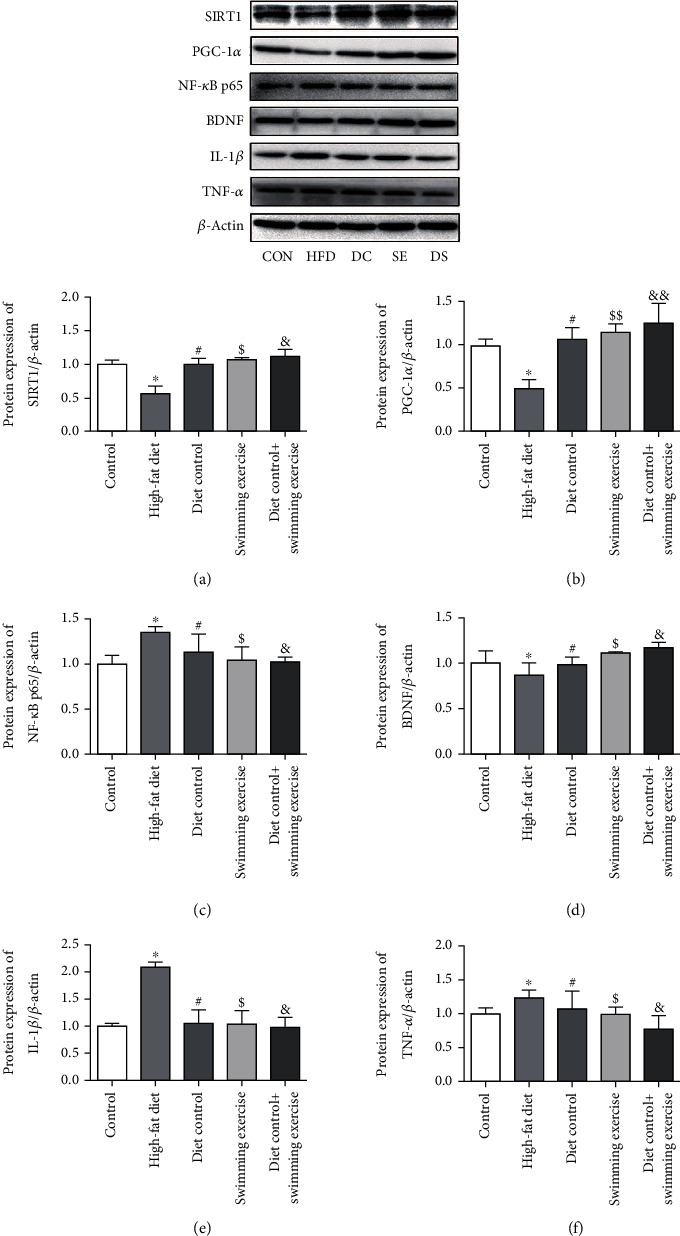
The expression of SIRT1, NF-*κ*B p65, IL-1*β*, TNF-*α*, PGC-1*α*, and BDNF proteins in the ApoE-/- mouse hippocampus. (a) The expression of SIRT1 in the hippocampi of ApoE-/- mice. (b) The expression of PGC-1*α* in the hippocampi of ApoE-/- mice. (c) The expression of NF-*κ*B p65 in the hippocampi of ApoE-/- mice. (d) The expression of BDNF in the hippocampi of ApoE-/- mice. (e) The expression of IL-1*β* in the hippocampi of ApoE-/- mice. (f) The expression of TNF-*α* in the hippocampi of ApoE-/- mice. ^∗^*p* < 0.05 vs. the control group. #*p* < 0.05, $*p* < 0.05, $$*p* < 0.01, &*p* < 0.05, and &&*p* < 0.01 vs. the HFD group.

To investigate the impact of diet control and/or swimming exercise on HFD-induced neuroinflammation, we first tested the expression of neuroinflammation cytokines (NF-*κ*B p65, IL-1*β*, and TNF-*α*) in the ApoE-/- mouse hippocampus. Western blot analysis showed NF-*κ*B p65, IL-1*β*, and TNF-*α* overexpression in the hippocampus of HFD-induced ApoE-/- mice compared to the CON group (*p* < 0.05), whereas the expression of NF-*κ*B p65, IL-1*β*, and TNF-*α* was significantly inhibited in the DC, SE, and DS groups (*p* < 0.05) (Figures 5(c), 5(e), and 5(f)). To further confirm the protective role of the SIRT1-mediated pathway on neuroinflammation, we explored SIRT1, PGC-1*α*, and BDNF expression. Western blot analysis showed that SIRT1, PGC-1*α*, and BDNF expression was significantly downregulated in the HFD group compared to the CON group (*p* < 0.05), and the expression of SIRT1, PGC-1*α*, and BDNF was increased in the DC, SE, and DS groups (*p* < 0.01, *p* < 0.05) (Figures 5(a), 5(b), and 5(d)). Therefore, in view of the data above, we speculated that SIRT1 activated by diet control and/or swimming exercise might exert a crucial role in anti-neuroinflammation related to downregulation of NF-*κ*B p65 and upregulation of PGC-1*α*/BDNF expression.

## 4. Discussion

Owing to changes in dietetic habits, a high-fat diet is recognized as the biggest health threat worldwide. These unhealthy lifestyles are characterized by excess calorie intake and low calorie expenditure, which contribute to an increased risk for chronic diseases, such as obesity, type 2 diabetes, atherosclerosis, ischaemic stroke, and cardiovascular disease [25, 26]. In addition, both the HFD-induced neuroinflammatory response and physical inactivity are closely related to poor cognitive performance [27, 28]. Compelling evidence has demonstrated that HFD-induced obesity results in brain inflammation and potentially leads to memory loss [29], and similar findings were observed in the present study. From our point of view, diet control and aerobic exercise are considered the most promising therapeutic options to improve memory deficits.

The hippocampus is located between the thalamus and medial temporal lobe in the brain and is primarily responsible for cognitive functions [30]. The hippocampus is vulnerable to the HFD-induced inflammatory response, and a HFD impairs the memory-consolidation process [31]. TNF-*α* and IL-1*β*, as well-known proinflammatory cytokines, were observed to be distinctly increased in the hippocampus of mice fed a HFD for 8 weeks [32]. Increasing evidence indicates that consumption of a HFD for more than one week is sufficient to generate learning and memory deficits and hippocampal plasticity impairment [33]. Furthermore, energy expenditure far below caloric intake under a sedentary lifestyle is the major contributor to obesity, which aggravates HFD-induced hippocampus-dependent memory loss [11]. TNF-*α* and IL-1*β* are confirmed to be the key regulators of synaptic plasticity and memory loss [34, 35]. As expected, higher levels of TNF-*α* and IL-1*β* were observed in the hippocampus of ApoE-/- mice in the HFD group, further suggesting that exposure to a HFD accelerated neuroinflammation in the hippocampus and resulted in cognitive dysfunction. Diet control and/or swimming exercise suppressed the neuroinflammatory response in the hippocampus and improved cognitive deficits.

Neuroinflammation affects the structure and function of the hippocampus. Microstructural changes in the hippocampus are one of the characteristic biological markers of cognitive deficits and impaired performance in learning memory [36]. DTI is a novel MRI-based imaging technique to detect early subtle alterations in white matter structure that are closely associated with the pathophysiology of cognitive impairment [37]. DTI can measure the direction and extent of the diffusion of water molecules in a three-dimensional space, reflecting the diffusion anisotropic characteristics of living tissues in white matter. Currently, DTI has been widely used to evaluate the white matter microstructure in individuals [38]. FA, a measure of the degree of directionality of water diffusion, is the main indicator of DTI [39]. Lower levels of FA in white matter generally imply reduced microstructural white matter integrity [40]. Decreased FA in the hippocampus corresponded to worse memory performance in older healthy individuals [41]. Our data revealed a remarkable decrease in FA in the bilateral hippocampus of HFD-fed ApoE-/- mice, indicating that the HFD promoted microstructural white matter integrity alterations in the hippocampus. However, microstructural alterations in the hippocampus and cognitive impairment were improved in mice with diet control and/or swimming exercise. This finding was consistent with accumulating evidence that diet control and/or aerobic exercise ameliorated HFD-induced cognitive decline by improving microstructural changes in the hippocampus.

MRS is a noninvasive imaging technique to detect and quantify metabolic changes in vivo. The main neurometabolites detected in MRS are NAA, Cho, MI, and Cr [42]. Aberrant metabolism was one of the features displayed before the occurrence of microstructural alterations in the brain [43]. It was reported that positive correlations of NAA/Cr and Cho/Cr with cognitive impairment and decreases in NAA/Cr and Cho/Cr were found in patients with MCI subsequently converting to AD [44]. It was demonstrated that metabolite abnormalities in the pathologic progression of AD were characterized by an increase in the MI/Cr ratio [45]. Therefore, progression in cognitive decline is accompanied by decreases in NAA/Cr and Cho/Cr and increases in MI/Cr. However, NAA is found almost exclusively in neurons and is recognized as a pivotal indicator of neuronal loss and dysfunction. A decreased concentration of NAA or a reduction in the NAA/Cr ratio reflects damage to neuronal microstructure and reduced neural metabolism, which are regarded as promoters of the occurrence of cognitive decline [46]. NAA is the marker of integrity maintenance in neurons, whereas MI is closely associated with CNS inflammation [47]. Our study revealed that lower levels of NAA/Cr and Cho/Cr and higher levels of MI/Cr were observed in the bilateral hippocampus of HFD-fed mice, and diet control and/or swimming exercise reversed this tendency, indicating that the HFD could impair hippocampal-dependent cognition. Diet control and/or swimming exercise improved cognition by affecting neurometabolites in the hippocampus.

SIRT1 has been confirmed to be involved in the regulation of neuroinflammation [48]. As an NAD^+^-dependent deacetylase, SIRT1 can deacetylate lysine residues on various substrates to regulate anti-inflammatory and neuroprotective effects, such as NF-*κ*B and PGC-1*α* [16]. NF-*κ*B, as a sensitive transcription factor in the inflammatory pathway, plays a pivotal role in neuroinflammatory responses. Activation of NF-*κ*B can regulate the release of TNF-*α* and IL-1*β* in the hippocampus, which is responsible for inflammatory diseases of the central nervous system [49]. NF-*κ*B is a family of transcriptional complexes composed of five constituent proteins: RelA (p65), RelB, c-Rel, NF-*κ*B1 (p50), and NF-*κ*B2 (p52) [50]. In addition, the members of NF-*κ*B have a C-terminal transactivation domain (TAD), including RelA (p65), RelB, and c-Rel. The predominant form of NF-*κ*B is a heterodimer of RelA (p65) and NF-*κ*B1 (p50) in the nucleus. SIRT1 can deacetylate lysine residue 310 on RelA (p65), which plays a dominant role in regulating cellular processes by binding to target genes [51]. SIRT1-mediated acetylation of RelA (p65) inhibits the transcriptional activity of NF-*κ*B, as well as the expression of TNF-*α* and IL-1*β* [21]. It has been confirmed that PGC-1*α* is a downstream target of SIRT1 [52]. PGC-1*α*, first described in the oxidative stress system, binds to numerous transcription factors involved in the regulation of oxidative metabolism and mitochondrial biogenesis [53]. There is compelling evidence that PGC-1*α* plays an essential role in the neuroprotective effect [54] and that swimming exercise can upregulate the expression of PGC-1*α* [55]. Overexpression of PGC-1*α* induced hippocampal BDNF expression, leading to improved cognitive impairment [56]. Exercise-induced PGC-1*α* was accompanied by an increase in BDNF release [57], indicating that swimming exercise could ameliorate cognitive decline through SIRT1/PGC-1*α*/BDNF. In this study, HFD increased the expression of NF-*κ*B p65, TNF-*α*, and IL-1*β* and inhibited SIRT1, PGC-1*α*, and BDNF expression in the hippocampus of ApoE-/- mice. However, diet control and/or swimming exercise increased the expression of SIRT1, PGC-1*α*, and BDNF and suppressed NF-*κ*B p65, TNF-*α*, and IL-1*β* expression in the hippocampus. Our findings indicated that diet control and/or swimming exercise improved HFD-induced cognitive impairment through antineuroinflammation, which was associated with activating the SIRT1-NF-*κ*B and SIRT1-PGC-1*α*-BDNF pathways.

## 5. Conclusions

In summary, our results further confirmed that HFD and low physical activity are regarded as threats to health and contribute to cognitive decline through neuroinflammation and microstructural changes in the hippocampus. Dietary control and physical activity ameliorate HFD-induced cognitive impairment and abnormal neurometabolism, which is associated with SIRT1-mediated NF-*κ*B pathways and PGC-1*α*-BDNF pathways.

## Data Availability

The experiment data are available from the corresponding author upon reasonable request.
